# Safety and efficacy of fluticasone/formoterol combination therapy in adolescent and adult patients with mild-to-moderate asthma: a randomised controlled trial

**DOI:** 10.1186/1471-2466-12-67

**Published:** 2012-10-18

**Authors:** Robert A Nathan, Anthony D’Urzo, Viktor Blazhko, Kirsten Kaiser

**Affiliations:** 1 Asthma and Allergy Associates PC, 2709 North Tejon Street, Colorado Springs, CO, USA; 2 Department of Family and Community Medicine (DFCM), University of Toronto, Ontario, Canada; 3 Kharkiv City Clinical Hospital #13, Kharkiv, Ukraine; 4 SkyePharma, Muttenz, Switzerland

## Abstract

**Background:**

This study investigated the efficacy and safety of a new asthma therapy combining fluticasone propionate and formoterol fumarate (fluticasone/formoterol; *flutiform*^®^), administered twice daily (b.i.d.) via a single aerosol inhaler, compared with its individual components administered separately and placebo, in patients with mild-to-moderate asthma.

**Methods:**

Patients aged ≥ 12 years were evenly randomised to 12 weeks of treatment with fluticasone/formoterol (100/10 μg b.i.d.), fluticasone (100 μg b.i.d.), formoterol (10 μg b.i.d.), or placebo, in this double-blind, parallel group, multicentre study. The three co-primary endpoints were: a) change in forced expiratory volume in the first second (FEV_1_) from morning pre-dose at baseline to pre-dose at week 12 for the comparison with formoterol; b) change in FEV_1_ from morning pre-dose at baseline to 2 hours post-dose at week 12 for the comparison with fluticasone, and c) time to discontinuation due to lack of efficacy from baseline to week 12 for the comparison with placebo. Safety was assessed based on adverse events, clinical laboratory tests and vital sign evaluations.

**Results:**

Statistically significant differences were demonstrated for all the three co-primary endpoints. Fluticasone/formoterol combination therapy showed significantly greater improvements from baseline to end of study in the change in pre-dose FEV_1_ compared with formoterol (Least Squares (LS) mean treatment difference: 0.101 L; 95% Confidence Interval (CI): 0.002, 0.199; p = 0.045) and the change in pre-dose compared with 2 hours post-dose FEV_1_ versus fluticasone (LS mean treatment difference: 0.200 L; 95% CI: 0.109, 0.292; p < 0.001). The time to discontinuation due to lack of efficacy was significantly longer for patients in the combination therapy group compared with those receiving placebo (p = 0.015). Overall, the results from multiple secondary endpoints assessing lung function, asthma symptoms, and rescue medication use supported the superior efficacy of the combination product compared with fluticasone, formoterol, and placebo. The fluticasone/formoterol combination therapy had a good safety and tolerability profile over the 12 week treatment period.

**Conclusions:**

Fluticasone/formoterol had a good safety and tolerability profile and showed statistically superior efficacy for the three co-primary endpoints compared to fluticasone, formoterol, and placebo, in adolescents and adults with mild-to-moderate asthma.

**EudraCT number:** 2007-002866-36; **US NCT number:** NCT00393991

## Background

Asthma is a chronic inflammatory disorder of the airways. It is associated with variable airflow obstruction related to airway hyperresponsiveness and bronchoconstriction. For persistent asthma, inhaled corticosteroids (ICSs) are recommended as one of the most effective treatments for airway inflammation. Nonetheless, for a significant number of patients symptoms persist and additional therapy is required [1-5].

Landmark studies in adult and adolescents have demonstrated that patients using ICS and long-acting β_2_-agonist (LABA) combination therapy achieved better asthma control compared to more than doubling the dose of ICS or administration of ICS in combination with other therapeutic agents [2,4-12], while further studies specifically in children and adolescents aged up to 16 years have reported at least similar efficacy with ICS/LABA compared with doubling the ICS dose [13,14]. In addition, research has also shown that the interactions between ICSs and LABAs potentiate each other’s respective therapeutic effects at the molecular level [15-18].

The ICS, fluticasone propionate (fluticasone), has a well-established safety and efficacy profile, and exerts a potent and sustained anti-inflammatory effect [19-22]. The LABA, formoterol fumarate (formoterol), has rapid, dose-dependent bronchodilatory effects, with an onset of action of 1 to 3 minutes [23], similar to salbutamol and faster than that of salmeterol [24-27]. Extensive research into the safety and efficacy of these two molecules is widely documented in the literature [19-30], and suggests that the fluticasone/formoterol combination may provide clinicians with a new and efficacious treatment for the management of persistent asthma.

The study presented here evaluated the efficacy and safety of fluticasone and formoterol combination therapy (fluticasone/formoterol; *flutiform*®), administered via a single aerosol inhaler, in adolescent and adult patients with mild-to-moderate asthma.

## Methods

### Study design

This was a 12-week, randomised, double-blind, placebo- and active-controlled, parallel-group study, conducted at 59 centres in North America and Europe. The study was conducted in accordance with ICH GCP and as per the ethical principles of the Declaration of Helsinki. The Institutional Review Boards or Independent Ethics Committees at each participating centre reviewed and approved the protocol (United States: Schulman Associates Institutional Review Board Inc., Cincinnati, Ohio; University of Florida Health Science Center IRB-01, Gainesville, Florida; Baylor Research Institute IRB, Dallas, Texas; Marywood University IRB, Scranton, Pennsylvania; Canada: IRB Services, Aurora, Ontario; Research Ethics Board MUHC-MGH Site, Montreal, Quebec; Europe: Ethics Commission of State Pharmacological Center of Health Ministry of Ukraine, Kiev, Ukraine). Written informed consent was obtained from all patients (or the parents or guardians of patients under 18 years of age) before they were enrolled into the study.

The efficacy and safety of fluticasone/formoterol combination therapy 100/10 μg, administered twice daily (b.i.d.) (50/5 μg, 2 inhalations b.i.d.) via a single hydrofluoroalkane (HFA) pressurised metered-dose inhaler (pMDI), was compared with the individual components administered separately (fluticasone, 100 μg b.i.d. pMDI [50 μg, 2 inhalations b.i.d.]; formoterol 10 μg b.i.d. pMDI [5 μg, 2 inhalations b.i.d.]), and placebo (pMDI [2 inhalations, b.i.d.]).

### Patients

Patients of both sexes, aged 12 years and over, with a history of asthma of at least 12 months prior to screening, as defined by the National Asthma Education and Prevention Program [31], were considered eligible for study enrolment. Eligible patients had either a documented history of ICS use for at least 4 weeks before screening, at a daily dose of not more than 500 μg fluticasone HFA pMDI (or equivalent), or were not on ICS therapy for at least 12 weeks prior to the screening visit. All patients were required to have a Forced Expiratory Volume in the first second (FEV_1_) between 60% and 85% (inclusive) of predicted normal values at both screening and baseline visits. Patients also needed to demonstrate FEV_1_ reversibility, i.e. reversible bronchoconstriction for patients who did not have a history of documented reversibility within the 12 months prior to the screening visit. These patients underwent a reversibility testing procedure, after the pulmonary function tests at screening, defined as a ≥14.5 % increase 15–30 minutes following albuterol/salbutamol aerosol inhalation (200 to 400 μg, as appropriate, i.e. two inhalations of 100 μg, separated by a period of 1 minute. If reversibility was not met, FEV_1_ was re-assessed within another 30 minutes, and if still not met, two further inhalations of albuterol/salbutamol were administered and reversibility was re-assessed). All patients had to be able to demonstrate satisfactory aerosol technique and accurate use of the telephone diary system.

Patients were excluded from the study if they had a history of life-threatening asthma within the previous 12 months or during the run-in period. Patients with a history of systemic corticosteroid use within the previous 3 months, omalizumab use within the previous 6 months, or leukotriene antagonist use within the week before screening, were also excluded. Other exclusion criteria included significant, non-reversible pulmonary disease, respiratory tract infections within the 4 weeks prior to screening visit or during the run-in period, significant medical illness, a smoking history of at least 10 pack-years or current smoking history within the previous year, and hypersensitivity to study medication. Patients were also excluded if they had received β-blockers, tricyclic antidepressants, monoamine oxidase inhibitors, quinidine-type antiarrhythmics, or drugs known to inhibit CYP3A4, within the week prior to screening. However, use of a LABA prior to screening was permitted.

### Interventions

The run-in period was used to confirm that all patients were symptomatic and to ensure that the baseline assessments were standardised across all patients after discontinuing their respective asthma medications. For patients who were ICS-requiring prior to screening, the run-in period lasted 14 ± 3 days during which time they received fluticasone (pMDI; 50 μg b.i.d.) as maintenance therapy. For patients with no history of ICS use, the run-in lasted between 14 to 28 days and they received no maintenance therapy during this time. Rescue medication was available to all patients for deteriorating asthma symptoms. During any 7 consecutive days of the run-in, patients were required to use at least two inhalations per day of rescue albuterol/salbutamol medication for at least 3 days and to have either 3 or more days with asthma symptoms or one night with sleep disturbance due to asthma. At the baseline visit, which was defined as week 0 and followed the run-in period, patients returned to the study site to complete the randomisation procedures (assessment of pulmonary function and general asthma symptom-based endpoints) and to confirm that randomisation criteria were met.

**Figure 1:**
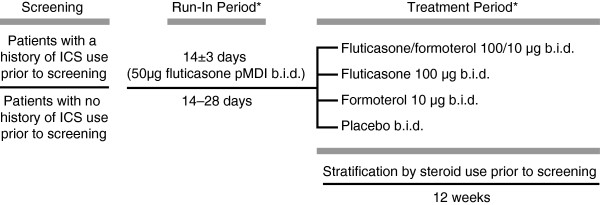
**Study design.** *Albuterol/salbutamol *pro re nata* (as needed) as rescue medication. b.i.d. = twice daily; ICS = inhaled corticosteroid; pMDI = pressurised metered dose inhaler.

At the end of the run-in period, eligible patients were randomised equally into one of the following four blinded treatment arms using minimisation with biased coin assignment [32], stratified according to prior steroid use, study site, and the subgroup of patients aged 12 to 18 years. Patients were provided with two inhalers: one for fluticasone/formoterol, formoterol or placebo (which were identical in appearance), and one for fluticasone or a visually identical fluticasone placebo. Study medication was administered twice daily for 12 weeks, taking two actuations from each device twice daily (8 inhalations per day): fluticasone/formoterol 100/10 μg (50/5 μg, 2 inhalations b.i.d.) and placebo b.i.d., fluticasone 100 μg (50 μg, 2 inhalations b.i.d.) and placebo b.i.d., formoterol 10 μg (5 μg, 2 inhalations b.i.d.) and placebo b.i.d., or placebo (2 inhalations, 2 devices, b.i.d.) (Figure 1). All study medications were administered via a pMDI without the use of a spacer. Patients were required to have a 1-minute interval between inhalations, always use the pMDIs in the same sequence and rinse their mouth thoroughly after dosing. All other asthma medications were prohibited during the study, except for albuterol/salbutamol, the use of which was permitted, as needed, in case of worsening asthma symptoms. An Interactive Voice Response System was used for patient enrolment, treatment allocation, and generation of patient identification number. The use of dummy placebo inhalers ensured that blinding was maintained throughout the study. The investigators, study site personnel, and representatives involved in monitoring, data management, any other aspect of the study, including sponsor personnel, were blinded throughout the study. Treatment assignment was strictly confidential and accessible only to authorised persons until the time of unblinding.

Patient adherence to assigned study medication regimen was assessed based on the data recorded via a telephone diary system. Each patient recorded the number of actuations of study and rescue medication they had used during both the run-in and the treatment periods. A safety follow-up was carried out two weeks after last dose of study medication by telephone.

### Efficacy assessments

The efficacy of fluticasone/formoterol combination therapy in comparison with fluticasone, formoterol, and placebo was evaluated using three co-primary endpoints: the mean change in FEV_1_ (as measured in the clinic) from pre-dose at baseline to pre-dose at week 12 (a comparison of fluticasone/formoterol versus formoterol alone) was used to assess the contribution of the antiinflammatory component from the fluticasone/formoterol combination; the mean change in FEV_1_ (in clinic) from pre-dose at baseline to 2 hours post-dose at week 12 (a comparison of fluticasone/formoterol versus fluticasone alone) was used to assess the contribution of the bronchodilator component from the combination product, and discontinuation due to lack of efficacy was used to evaluate the efficacy of the fluticasone/formoterol combination compared with placebo. Lack of efficacy was defined by asthma exacerbations and loss of asthma control (see below for definitions), and these two classifications were combined for the analysis. In order to demonstrate superior efficacy, fluticasone/formoterol therapy had to achieve statistical significance over the relevant comparator treatments for each of the three co-primary endpoints.

Secondary efficacy endpoints comprised additional pulmonary function tests including FEV_1_ % predicted normal, Forced Vital Capacity (FVC), frequency of asthma exacerbations, and data gathered from patients’ telephone diaries including morning and evening Peak Expiratory Flow Rate (PEFR), asthma symptom scores, sleep disturbance scores, and frequency of rescue medication use.

FEV_1_ was measured in the clinic at baseline and at weeks 2, 4, 8, and 12 by spirometry in accordance with the American Thoracic Society/European Respiratory Society Task Force guidelines [33]. Predicted FEV_1_ values were determined using the values of Polgar and Promadhat [34] for patients aged 12–17 years, and those of Crapo *et al.*[35] for patients aged 18 years and older. Spirometry values were also adjusted for race. PEFR was measured twice daily, pre-dose, by means of a MicroPeak peak flow meter (Micromedical, Chatham Maritime, Kent, UK), and patients recorded the results using the telephone diary system.

The frequency and severity (defined as either ‘mild-to-moderate’ or ‘severe’) of asthma exacerbations were recorded throughout the study. Mild-to-moderate exacerbations were defined as any of the following occurring for at least 2 consecutive days: i) pre-dose PEFR measurements more than 30% below the values measured at baseline, or ii) awakening during the night because of asthma, or iii) the use of additional rescue medication of more than three inhalations per day compared with baseline. Severe exacerbations were defined as the deterioration in asthma that required additional therapy (for example, systemic steroids), or an emergency visit or hospitalisation due to asthma.

Asthma symptoms, scored on a six-point scale ranging from 0 to 5 (0 = no symptoms; 5 = asthma so severe that the patient was unable to go to work or school or to carry out normal daily activities), and sleep disturbances, scored on a five-point scale ranging from 0 to 4 (0 = slept through the night, no asthma; 4 = could not sleep at all because of asthma), were recorded via the telephone diary system.

Patients were withdrawn from the study because of lack of treatment efficacy (asthma exacerbations and loss of asthma control) if any of the following five criteria were met: i) a severe asthma exacerbation requiring emergency treatment, hospitalisation, or use of any asthma medication not permitted in the study protocol, ii) a decrease in pre-dose FEV_1_ (as measured in the clinic) of more than 20% from baseline, iii) a decrease in morning pre-dose PEFR (from telephone diaries) of more than 25% from baseline on more than 3 of the 7 days before a study visit, iv) excessive use (more than 12 actuations per day) of rescue medication on more than 3 of the 7 days before a study visit, or v) nocturnal awakening due to asthma, that required rescue medication, on more than 2 of 7 days before a study visit.

### Safety assessments

Safety assessments were carried out throughout the study based on adverse events reported, vital signs, a 12-lead electrocardiogram (ECG), and clinical laboratory testing.

### Statistical analyses and sample size calculation

Efficacy analyses were performed on the full analysis set (FAS) (all patients who received at least one dose of study medication, had a baseline FEV_1_ measurement and at least one post-baseline pre-dose and 2-hour post-dose FEV_1_ measurement), the per-protocol (PP) population (all patients in the FAS who did not have a major protocol violation, which included patients who did not take study medication on at least 50% of the days that the patient was in the study or if the patient did not return for two study visits in a row), and the safety population (all randomised patients who received at least one inhalation of study medication).

The change in morning pre-dose FEV_1_ from baseline to pre-dose at weeks 2, 4, 8, and 12, and change in morning pre-dose FEV_1_ from baseline to 2-hours post-dose at weeks 2, 4, 8, and 12 were compared between the treatment groups using analysis of covariance (ANCOVA), with treatment group, centre, and previous steroid use as main effects, and baseline FEV_1_ as a continuous covariate. Missing data were replaced using the last observation carried forward (LOCF) approach. To analyse the time to discontinuation due to lack of efficacy, a stratified log-rank test was performed adjusting for treatment group and previous steroid use. In this superiority analysis (which used a two-sided t-test), superiority for each the three co-primary endpoints was confirmed if the lower limit of the 95% confidence interval (CI) for the between-treatment difference did not span 0, and hence *p* < 0.05. There was no requirement for the CI to be above a pre-defined threshold.

For the secondary endpoints, the differences between groups for the change from baseline in PEFR, asthma symptom scores, sleep disturbance scores, and rescue medication use were analysed using the ANCOVA model described above, with the relevant baseline value as the continuous covariate. Differences in the frequency of exacerbations between groups were tested by logistic regression analysis with effects for treatment group and previous steroid use, and differences between groups for the percentage of days with an asthma exacerbation and percentage of asthma control days were assessed using van Elteren’s method for combining Wilcoxon rank sum test results from independent strata, with prior steroid use as the stratum for the analysis.

Provided all three co-primary endpoints were statistically significant, the secondary endpoints were then evaluated using a sequential gatekeeper approach [36] for the three treatment comparisons, according to the following order: i) fluticasone/formoterol combination therapy versus placebo, and ii) fluticasone/formoterol combination therapy versus fluticasone alone and fluticasone/formoterol combination therapy versus formoterol alone.

The first four secondary endpoints were analysed firstly for combination therapy vs. placebo, in the following order, based on the mean change from baseline to week 12: morning PEFR, evening PEFR, use of rescue medication, and asthma symptom scores. Provided that each of these analyses returned statistically significant results for combination product vs. placebo (p < 0.05), the subsequent analyses (combination therapy vs. fluticasone alone and vs. formoterol alone) could then be carried out in the same order. Statistical analyses were two-sided and significance was measured at the 0.05α level. If both tests were significant at the 0.05α level, the next endpoint could be evaluated for confirmatory statistical significance. If one of the two tests was not significant at the 0.05α level, for example the analysis of morning PEFR for combination therapy vs. fluticasone alone, the other test (morning PEFR for combination product vs. formoterol alone) could be evaluated for statistical significance at the 0.025α level, however all formal testing of the remaining secondary endpoints was suspended (i.e. for both combination product vs. fluticasone and vs. formoterol). If the analyses were not statistically significant for the combination product versus either comparator then, once again, all remaining confirmatory sequential testing was formally suspended. If the sequential gatekeeper approach for the three comparative tests was statistically significant for each of the four endpoints, confirmatory sequential testing of the remaining secondary endpoints was carried out in the following order, using the same Hochberg methodology as described above: percentage of symptom-free days (defined as days with an asthma symptom score of zero), percentage of rescue medication-free days (days with no use of rescue medication), percentage of asthma control days (days with asthma symptom score of zero, sleep disturbance score of zero, and no use of rescue medication), the proportion of patients with treatment-emergent asthma exacerbations, sleep disturbance scores, and the percentage of awakening-free nights (nights with a sleep disturbance score of zero). Pre-specified subgroup analyses were performed for all three co-primary endpoints based on prior ICS use, using an ANCOVA, with age and study site as factors, and using the LOCF approach on the FAS.

Safety analyses were performed for all randomised patients who received at least one inhalation of study medication (the safety population).

For pre-dose or 2-hours post-dose FEV_1_ measures, a sample size of 92 patients per treatment group in the study would have 85% power to detect a significant difference between two treatment groups using a two-sided *t*-test with α=0.05, assuming a difference of 0.2 L with respect to mean change from morning pre-dose baseline to either morning pre-dose FEV_1_ at week 12 or 2-hour post-dose FEV_1_ at week 12, and a common standard deviation (SD) of 0.45. It was therefore planned to enrol 108 patients in each group to account for an approximately 15% drop out rate. Assuming that 10% of fluticasone/formoterol and 30% of placebo group patients would discontinue due to lack of efficacy, with 92 patients per treatment group there would be 90% power to detect this difference using a two-sided log-rank test with α = 0.05.

## Results

**Figure 2:**
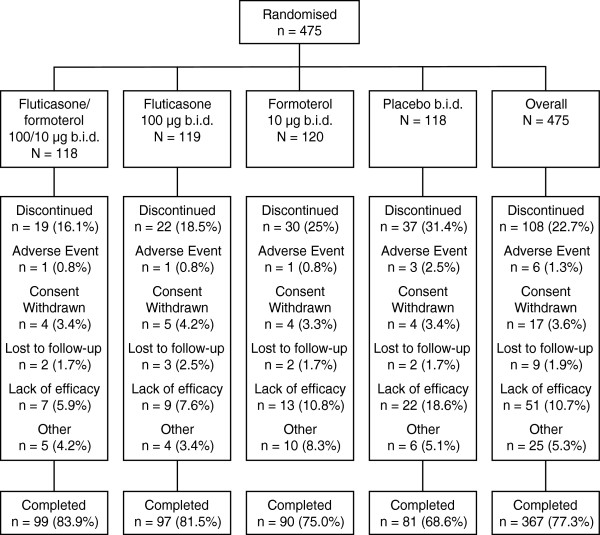
Patient flow diagram.

A total of 475 patients were randomised to treatment, including 33 adolescents (6.9%). Of the 475 patients, 333 took part at sites based in the United States, 80 were based in Canada, and 62 in the Ukraine, and, overall, 367 (77.3%) patients completed the study (Figure 2). Treatment groups were well-matched with regard to demographics and baseline characteristics, with little difference between groups with respect to lung function reversibility. Prior to screening, a total of 29.4% of patients had received ICS monotherapy, and 20.0% had received combined ICS and LABA combination therapy (Table 1). The median FEV_1_ % predicted value at baseline ranged from 72.0 to 75.0 (Table 1). Mean compliance rates ranged from 84% to 85% across treatment arms. There were 459 randomised patients in the FAS (115, 117, 116, and 111 in the combination, fluticasone, formoterol, and placebo groups, respectively); 408 in the PP population (103 in each of the combination and fluticasone groups, and 101 in each of the formoterol and placebo groups); all 475 patients were in the safety population.

**Table 1:** Patient baseline demographic and asthma characteristics, Full Analysis Set

**Characteristic**	**Treatment group**				
	**Fluticasone/formoterol 100/10 μg b.i.d. N = 115**	**Fluticasone 100 μg b.i.d. N = 117**	**Formoterol 10 μg b.i.d. N = 116**	**Placebo b.i.d. N = 111**	**Overall N = 459**
**Gender, n (%)**					
Female	72 (62.6)	71 (60.7)	63 (54.3)	70 (63.1)	276 (60.1)
Male	43 (37.4)	46 (39.3)	53 (45.7)	41 (36.9)	183 (39.9)
**Ethnic origin, n (%)**					
White/Caucasian	89 (77.4)	88 (75.2)	83 (71.6)	79 (71.2)	339 (73.9)
Black	13 (11.3)	16 (13.7)	21 (18.1)	22 (19.8)	72 (15.7)
Asian	6 (5.2)	4 (3.4)	5 (4.3)	4 (3.6)	19 (4.1)
Hispanic	6 (5.2)	8 (6.8)	6 (5.2)	5 (4.5)	25 (5.4)
Other	1 (0.9)	1 (0.9)	1 (0.9)	1 (0.9)	4 (0.9)
**Age, years**					
Mean (SD)	39.8 (14.54)	38.3 (14.45)	39.1 (15.26)	38.1 (13.67)	38.8 (14.47)
**Age categories, n (%)**					
12 to 17 years	7 (6.1)	9 (7.7)	9 (7.8)	6 (5.4)	31 (6.8)
≥ 18 years	108 (93.9)	108 (92.3)	107 (92.2)	105 (94.6)	428 (93.2)
**Steroid use, n (%)**					
Free^a^	59 (51.3)	60 (51.3)	58 (50.0)	55 (49.5)	232 (50.5)
Requiring^b^	56 (48.7)	57 (48.7)	58 (50.0)	56 (50.5)	227 (49.5)
**Prior ICS and ICS/LABA use, n (%)**					
ICS only	31 (27.0)	39 (33.3)	30 (25.9)	35 (31.5)	135 (29.4)
ICS and LABA	25 (21.7)	18 (15.4)	28 (24.1)	21 (18.9)	92 (20.0)
**Duration of asthma, years**^c^					
Mean (SD)	18.9 (13.40)	20.6 (13.84)	20.3 (14.48)	21.4 (12.83)	20.3 (13.64)
**FEV**_1_ % predicted^d^ at baseline^e^					
Mean (SD)	73.2 (7.54)	73.5 (8.14)	73.2 (7.79)	72.0 (7.97)	73.0 (7.86)
Median	72.0	75.0	73.0	72.0	73.0
FEV_1_ at baseline^e^, L					
Mean (SD)	2.416 (0.5790)	2.425 (0.6625)	2.459 (0.6231)	2.352 (0.6114)	2.414 (0.6192)
Median	2.370	2.330	2.375	2.250	2.340
**Reversibility at screening, %**					
	n = 114	n = 117	n = 116	n = 111	n = 458
Mean (SD)	23.2 (10.1)	22.8 (9.0)	21.8 (8.4)	22.8 (8.3)	22.6 (9.0)
Median	19.3	19.5	18.7	20.0	19.2

N = total number of patients; n = number of patients in specified category; SD = standard deviation; b.i.d. = twice daily; ICS = inhaled corticosteroids; LABA = long acting beta agonist; FEV_1_ = Forced Expiratory Volume in the first second.

a. Patient with no history of steroid use for at least 12 weeks prior to the screening visit.

b. Patient who used an inhaled steroid regimen at a dose not greater than 500 μg/day fluticasone (or equivalent steroid) for at least 4 weeks prior to screening.

c. Duration of asthma calculated as (Date of screening visit from Demographics CRF - Asthma diagnosis date)/ 365.25 and rounded to 1 decimal place.

d. Based on standardised predicted FEV_1_ values.

e. Baseline was the last available value prior to dosing at the baseline/week 0 visit.

### Primary efficacy endpoints

The three co-primary endpoints demonstrated superior efficacy of fluticasone/formoterol combination therapy compared to fluticasone, formoterol, and placebo, respectively (Table 2).

**Table 2:** **Mean change in FEV**_**1**_**(L) from pre-dose at baseline to pre-dose and 2-hour post-dose at week 12, (LOCF), time to discontinuation due to lack of efficacy and study duration for each treatment group, Full Analysis Set**

	**Treatment group**			
	**Fluticasone/formoterol 100/10 μg b.i.d. N = 115**	**Fluticasone 100 μg b.i.d. N = 117**	**Formoterol 10 μg b.i.d. N = 116**	**Placebo b.i.d. N = 111**
Baseline FEV_1_ (L)				
Mean (SD)	2.416 (0.5790)	2.425 (0.6625)	2.459 (0.6231)	2.352 (0.6114)
Change in FEV_1_ from pre-dose at baseline to pre-dose at week 12				
LS Mean (SE)	0.195 (0.038)	0.092 (0.037)	0.094 (0.038)	0.047 (0.037)
Difference from fluticasone/formoterol 100/10 μg b.i.d.: contribution from fluticasone component				
LS Mean (SE)		0.103 (0.050)	0.101 (0.050)	0.147 (0.051)
95% CI		0.005, 0.201	0.002, 0.199	0.048, 0.247
*p*-value		0.040	0.045	0.004
Change in FEV_1_ from pre-dose at baseline to 2 hours post-dose at week 12				
LS Mean (SE)	0.392 (0.035)	0.191 (0.034)	0.330 (0.035)	0.124 (0.035)
Difference from fluticasone/formoterol 100/10 μg b.i.d.: contribution from formoterol component				
LS Mean (SE)		0.200 (0.047)	0.062 (0.047)	0.267 (0.047)
95% CI		0.109, 0.292	−0.030, 0.153	0.175, 0.360
*p*-value		< 0.001	0.187	< 0.001
Discontinuation due to lack of efficacy				
Number, %	7 (6.1)	9 (7.7)	13 (11.2)	18 (16.2)
Time to discontinuation, weeks^a^				
Mean	6.9	4.7	4.6	5.5
*p*-value ^b^	0.015			

b.i.d. = twice daily; N = total number of patients; SD = standard deviation; LS = least squares; SE = standard error; NA = not applicable; CI = confidence interval; LOCF = last observation carried forward; FEV_1_ = Forced Expiratory Volume in the first second.

a. Time to discontinuation due to lack of efficacy calculated as (Date of early discontinuation - Date of first study drug administration+1)/7 and rounded to 1 decimal place (based on all patients who discontinued).

b. *p*-value based on stratified log-rank test adjusting for prior steroid use for fluticasone/formoterol 100/10 μg b.i.d. versus placebo.

**Figure 3:**
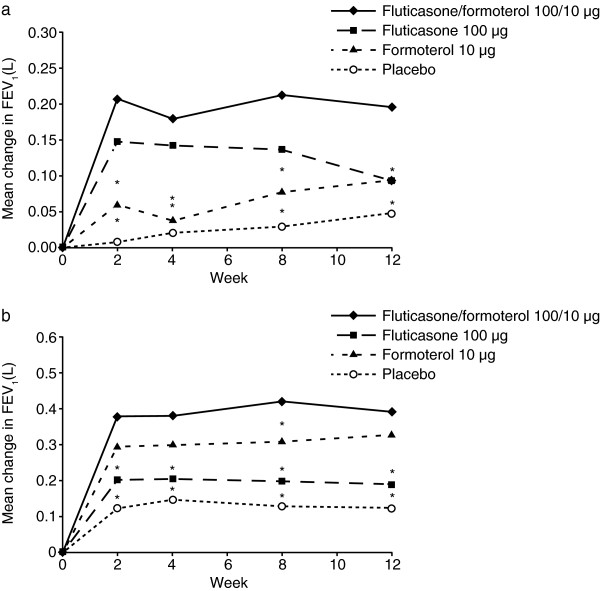
**A – Mean change in FEV**_**1**_**(L): mean change from baseline to pre-dose at weeks 2, 4, 8, and 12, Full Analysis Set (LOCF).** * P-value ≤ 0.05 versus fluticasone/formoterol 100/10 μg b.i.d. combination therapy treatment group. Baseline means were 2.416 L, 2.425 L, 2.459 L, and 2.352 L for the fluticasone/formoterol, fluticasone, formoterol, and placebo treatment groups, respectively, for all patients in the Full Analysis Set. b.i.d. = twice daily; FEV_1_ = forced expiratory volume in the first second; LOCF = last observation carried forward. **Figure**3**B – Mean change in FEV**_**1**_**(L): mean change from baseline to 2 hours post-dose at weeks 2, 4, 8, and 12, Full Analysis Set (LOCF).** * P-value ≤ 0.05 versus fluticasone/formoterol 100/10 μg b.i.d. combination therapy treatment group. Baseline means were 2.416 L, 2.425 L, 2.459 L, and 2.352 L for the fluticasone/formoterol, fluticasone, formoterol, and placebo treatment groups, respectively, for all patients in the Full Analysis Set. b.i.d. = twice daily; FEV_1_ = forced expiratory volume in the first second; LOCF = last observation carried forward.

The fluticasone/formoterol combination showed clinically relevant improvements in FEV_1_ from pre-dose at baseline to pre-dose and 2-hour post-dose at week 12 (Table 2). Furthermore, the contribution of the fluticasone component in the combination product, as analysed by the mean change in FEV_1_ from pre-dose at baseline to pre-dose at week 12, demonstrated statistically significant improvements for patients in the combination therapy treatment arm compared with those administered formoterol alone (LS mean difference = 0.101 L; 95% CI: 0.002, 0.199; p = 0.045). Similarly, the contribution of the formoterol component of the combination product, as analysed by the mean change in FEV_1_ from pre-dose at baseline to 2 hours post-dose at week 12, demonstrated statistically significant improvements for patients in the combination therapy treatment arm compared with those administered fluticasone alone (LS mean difference = 0.200 L; 95% CI: 0.109, 0.292; p < 0.001) (Table 2). The improvements in pre-dose FEV_1_ (Figure 3A) and 2-hour post-dose FEV_1_ (Figure 3B) with fluticasone/formoterol were demonstrated throughout the entire treatment period as shown by pulmonary function tests carried out at Weeks 2, 4, 8, and 12. Secondary analyses also showed that fluticasone/formoterol provided significantly greater improvements than fluticasone alone in FEV_1_ from pre-dose at baseline to pre-dose at week 12, and numerically greater improvements in FEV_1_ from pre-dose at baseline to 2 hours post-dose at week 12 compared with formoterol alone (Table 2).

Fluticasone/formoterol combination therapy was also shown to be superior to placebo with respect to the time to discontinuation due to lack of efficacy (due to either asthma exacerbation or to loss of asthma control) (log-rank p = 0.015) (Table 2). Furthermore, fewer patients discontinued due to lack of efficacy in the combination therapy group (6.1%) compared with those in the fluticasone group (7.7%), formoterol group (11.2%) or the placebo group (16.2%) (Table 2).

### Secondary efficacy endpoints

The secondary efficacy endpoints evaluated lung function, disease control and asthma symptoms. Overall, all of these evaluations supported the superior efficacy of fluticasone/formoterol combination therapy compared with the individual components and placebo. The combination product demonstrated numerically greater improvements for a number of the secondary endpoint evaluations versus all three of the comparators, with many endpoints meeting the criteria for statistical significance as per the sequential gatekeeping approach.

**Figure 4:**
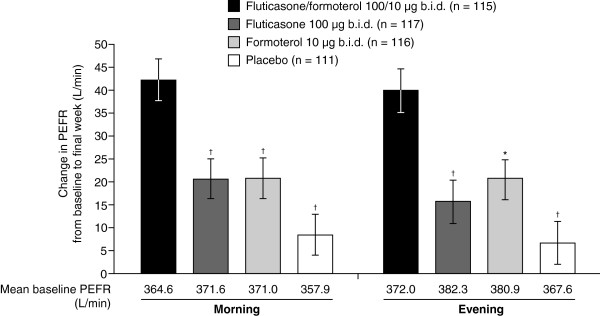
**Morning and evening PEFR (L/min): mean change from baseline to week 12, Full Analysis Set.** * P-value < 0.01 versus fluticasone/formoterol 100/10 μg b.i.d. combination therapy treatment group. † P-value < 0.001 versus fluticasone/formoterol 100/10 μg b.i.d. combination therapy treatment group. b.i.d. = twice daily; PEFR = peak expiratory flow rate; SE = standard error. Changes from baseline are shown as least-squares mean ± SE for the full analysis set.

The mean increase in morning and evening PEFR values from baseline to week 12 was statistically significantly greater (p < 0.01) for patients on the combination product compared with those administered fluticasone, formoterol or placebo (Figure 4).

Disease control, as evaluated by asthma control days, rescue medication-free days, symptom-free days, and awakening-free nights (Table 3), demonstrated numerically greater improvements for fluticasone/formoterol compared to all the comparator treatments. However, significant inferential statistical testing was only exploratory based on the sequential gatekeeping approach. Patients administered fluticasone/formoterol 100/10 μg b.i.d. demonstrated a five-fold increase in the percent of asthma control days from baseline (12.8%) to week 12 (69.1%), corresponding to 0.9 days per week at the start of the study compared to 4.8 days by the end of treatment. The mean increase in percent of asthma control days was 56.3% for the combination product, 44.0% for the fluticasone, 41.9% for the formoterol, and 36.0% for the placebo groups.

**Table 3:** Asthma control days (%), rescue medication-free days (%), symptom-free days (%), and awakening-free nights (%): mean change from baseline to week 12, Full Analysis Set

**Characteristic**	**Treatment group**			
	**Fluticasone/formoterol 100/10 μg b.i.d. N = 115**	**Fluticasone 100 μg b.i.d. N = 117**	**Formoterol 10 μg b.i.d. N = 116**	**Placebo b.i.d N = 111**
**Asthma control days (%)**	n = 109	n = 114	n = 112	n = 105
Baseline^a^, mean (SD)	12.8 (20.14)	14.3 (22.62)	11.5 (19.21)	10.0 (18.11)
Week 12, mean (SD)	69.1 (37.69)	58.3 (42.02)	53.4 (40.11)	46.0 (41.22)
Change to week 12				
Mean (SD)	56.3 (39.11)	44.0 (39.49)	41.9 (42.13)	36.0 (39.27)
Difference from fluticasone/formoterol 100/10 μg b.i.d.^b^				
*p*-value*		0.017†	0.117	0.012†
**Rescue medication-free days (%)**	n = 112	n = 116	n = 115	n = 109
Baseline^a^, mean (SD)	21.8 (24.38)	21.4 (25.55)	19.5 (24.51)	17.2 (20.14)
Week 12, mean (SD)	77.7 (32.12)	64.8 (39.49)	61.4 (37.19)	56.6 (39.95)
Change to week 12				
Mean (SD)	55.9 (36.43)	43.3 (37.69)	41.9 (39.49)	39.4 (38.69)
Difference from fluticasone/formoterol 100/10 μg b.i.d.^b^				
* p*-value*		0.020†	0.125	0.012†
**Symptom-free days (%)**	n=110	n=114	n=115	n=108
Baseline^a^, mean (SD)	28.0 (26.72)	28.6 (29.97)	22.6 (27.01)	22.7 (27.75)
Week 12, mean (SD)	77.4 (35.21)	65.9 (38.59)	60.5 (38.67)	58.3 (38.99)
Change to week 12				
Mean (SD)	49.4 (38.17)	37.3 (39.79)	38.0 (42.64)	35.6 (42.18)
Difference from fluticasone/formoterol 100/10 μg b.i.d.^b^				
* p*-value*		0.027†	0.195	0.151
**Awakening-free nights (%)**	n = 112	n = 116	n = 115	n = 108
Baseline^a^, mean (SD)	59.1 (33.79)	62.1 (33.73)	62.9 (34.51)	56.3 (37.40)
Week 12, mean (SD)	87.9 (26.73)	87.5 (26.77)	82.6 (31.57)	77.2 (34.17)
Change to week 12				
Mean (SD)	28.8 (33.91)	25.4 (35.92)	19.6 (35.68)	20.9 (41.05)
Difference from fluticasone/formoterol 100/10 μg b.i.d.^b^				
* p*-value*		0.790	0.055	0.052

b.i.d. = twice daily; N = total number of patients; n = number of patients in treatment group; FAS = full analysis set; SD = standard deviation.

a. Baseline was the 7-day average calculated on the last 7 days prior to the first dose of study drug.

b. Analysis method was Cochran-Mantel-Haenszel using van Elteren’s method for combining Wilcoxon rank sum test results from independent strata, with previous steroid use and site as the strata for the analysis.

*All *p* values were exploratory.

† p ≤ 0.050 versus fluticasone/formoterol 100/10 μg b.i.d. combination therapy but not statistically significant as per the sequential gatekeeping approach.

Overall, a lower percentage of patients on combination therapy experienced any asthma exacerbation (20.0%) compared to those administered the monotherapies (23.9% on fluticasone; 28.4% on formoterol) or placebo (32.4%), although the differences did not reach statistical significance. For patients in the fluticasone/formoterol group, 2.6% experienced a severe exacerbation, compared to 3.4% on fluticasone, 6.9% on formoterol, and 9.0% of patients on placebo (p = 0.048 for placebo vs fluticasone/formoterol).

Similarly, a larger mean increase in rescue medication-free days was observed in the combination therapy arm than in any of the comparator groups. In the fluticasone/formoterol group, a greater than three-fold increase was seen in the number of rescue medication-free days from baseline (21.8%) to week 12 (77.7%), corresponding to an improvement from 1.5 days/week to 5.4 days/week. Overall, the mean increase in percent rescue medication-free days was 55.9% in the combination therapy group compared to 43.3%, 41.9%, and 39.4% for the fluticasone, formoterol, and placebo groups, respectively (Table 3).

The mean percentage of symptom-free days at week 12 (77.4%, corresponding to 5.4 days/week) in the combination therapy group was 2.5-fold than seen at baseline (28.0%, corresponding to 2.0 days/week). The mean increase in symptom-free days in this group was 49.4%, compared with 37.3%, 38.0% and 35.6% in the fluticasone, formoterol and placebo groups, respectively.

This trend was also seen in the number of awakening-free nights for patients in the fluticasone/formoterol group. The mean percentage of awakening-free nights was approximately 1.5-fold greater by week 12 (87.9%) compared to baseline (59.1%), which corresponds to 4.1 awakening-free nights at baseline and 6.2 awakening-free nights at the end of study. The mean increase was 28.8% for patients in the combination therapy group compared to 25.4%, 19.6%, and 20.9% for patients in the fluticasone, formoterol, and placebo groups, respectively.

Asthma symptoms, as evaluated by rescue medication use, asthma symptom scores, and sleep disturbance scores, recorded daily by the patients, demonstrated numerically greater improvement for those administered fluticasone/formoterol compared to the individual components and placebo. Rescue medication use was statistically significant for the combination versus all three comparator groups, as evaluated using the sequential gatekeeping approach (Table 4).

**Table 4:** Use of rescue medication (number of inhalations/day), asthma symptom scores, and sleep disturbance scores: mean change from baseline to week 12, Full Analysis Set

**Characteristic**	**Treatment group**			
	**Fluticasone/formoterol 100/10 μg b.i.d. N = 115**	**Fluticasone 100 μg b.i.d. N = 117**	**Formoterol 10 μg b.i.d. N = 116**	**Placebo b.i.d. N = 111**
**Rescue medication use (inhalations/day)**	n = 112	n = 116	n = 115	n = 109
Baseline^a^, mean (SD)	2.8 (2.05)	3.0 (2.24)	3.0 (2.07)	3.0 (1.68)
Change to week 12				
Mean (SE)^b^	−2.22 (0.165)	−1.64 (0.160)	−1.62 (0.163)	−1.16 (0.162)
Difference from fluticasone/formoterol 100/10 μg b.i.d.^b^				
LS Mean (SE)		−0.58 (0.217)	−0.60 (0.218)	−1.06 (0.222)
95% CI		−1.01, -0.15	−1.03, -0.17	−1.50, -0.63
* p*-value*		0.008**	0.006**	<0.001**
**Asthma symptom scores**	n = 110	n = 114	n = 115	n = 108
Baseline^a^, mean (SD)	1.0 (0.60)	1.0 (0.64)	1.1 (0.61)	1.1 (0.62)
Change to week 12				
LS Mean (SE)^b^	−0.72 (0.060)	−0.59 (0.058)	−0.54 (0.059)	−0.51 (0.059)
Difference from fluticasone/formoterol 100/10 μg b.i.d.^b^				
LS Mean (SE)		−0.13 (0.079)	−0.18 (0.079)	−0.21 (0.081)
95% CI		−0.29, 0.03	−0.33, -0.02	−0.37, -0.05
* p*-value*		0.100	0.027†	0.011**
**Sleep disturbance scores**	n = 112	n = 116	n = 115	n = 108
Baseline^a^, mean (SD)	0.5 (0.54)	0.5 (0.45)	0.4 (0.49)	0.6 (0.57)
Change to week 12				
LS Mean (SE)^b^	−0.36 (0.033)	−0.34 (0.032)	−0.28 (0.033)	−0.27 (0.033)
Difference from fluticasone/formoterol 100/10 μg b.i.d.^b^				
LS Mean (SE)		−0.02 (0.043)	−0.09 (0.044)	−0.10 (0.044)
95% CI		−0.11, 0.07	−0.17, 0.00	−0.18, -0.01
*p*-value*		0.632	0.053	0.031†

b.i.d. = twice daily; N = number of patients in treatment group; n = number of patients with data available; CI = confidence interval; FAS = full analysis set; LS = least squares; SD = standard deviation; SE = standard error.

a. Baseline was the 7-day average calculated in the last 7 days prior to the first dose of study drug.

b. LS mean, SE, CI and p-value are from ANCOVA with factors for treatment group, site, and prior steroid use, with baseline value as a continuous covariate.

* All *p*-values were exploratory.

** *p* ≤ 0.050 versus fluticasone/formoterol 100/10 μg b.i.d. combination therapy and statistically significant as per the sequential gatekeeping approach.

† *p* ≤ 0.050 versus fluticasone/formoterol 100/10 μg b.i.d. combination therapy but not statistically significant as per the sequential gatekeeping approach.

### Sub-group analyses

Pre-specified subgroup analyses were performed based on ICS use prior to study entry, although it should be acknowledged that this study was not powered to assess these endpoints statistically. For patients with no history of ICS use prior to study enrolment, the difference between the mean change in pre-dose FEV_1_ from baseline to week 12 between the combination therapy and formoterol groups was not statistically significant (LS mean treatment difference: 0.094 L; 95% CI: -0.050, 0.237; p *=* 0.200). However, the mean increase in FEV_1_ from pre-dose at baseline to 2 hours post-dose at week 12 was statistically significant in the combination therapy group compared with fluticasone alone (LS mean treatment difference: 0.212 L; 95% CI: 0.070, 0.354; p *=* 0.004).

For patients with a prior history of ICS use, the combination product was statistically significantly superior to each of the comparators for the mean change from pre-dose FEV_1_ at baseline to both pre-dose at week 12 (LS mean treatment difference combination product compared with formoterol alone: 0.139 L; 95% CI: 0.000, 0.277; p = 0.050) and 2 hours post-dose at week 12 (LS mean treatment difference combination product vs. fluticasone alone: 0.172 L; 95% CI: 0.054, 0.291; p = 0.005).

With respect to discontinuations due to lack of treatment efficacy, no statistically significant treatment group difference was identified among patients with no history of prior steroid use (log-rank p = 0.795); 4 patients (7.3%) from the combination therapy group and 3 (5.8%) from the placebo group discontinued prematurely. For patients with a history of ICS use, fluticasone/formoterol combination was demonstrated to be statistically significantly superior to placebo (p = 0.002, log-rank test); 3 patients (5.7%) receiving fluticasone/formoterol discontinued early compared to 15 patients (36.6%) administered placebo.

### Safety and tolerability

Combination therapy with fluticasone/formoterol was well tolerated. Adverse events were reported by 38 (32.2%) patients in the fluticasone/formoterol group, 47 (39.5%) patients in the fluticasone group, 44 (36.7%) patients in the formoterol group, and 46 patients (39.0%) in the placebo group (Table 5). No deaths or asthma exacerbations requiring hospitalisation were reported. Most adverse events were mild or moderate in severity. Only one serious adverse event occurred during the study, a case of right-sided renal colic in a 70-year-old male patient receiving fluticasone/formoterol therapy which was not considered by the Investigator to be treatment-related.

**Table 5:** Overview of Adverse Events, Safety Population

	**Treatment group**			
	**Fluticasone/formoterol 100/10 μg b.i.d. N = 118**	**Fluticasone 100 μg b.i.d. N = 119**	**Formoterol 10 μg b.i.d. N = 120**	**Placebo b.i.d. N = 118**
Any AE, n (%)	38 (32.2)	47 (39.5)	44 (36.7)	46 (39.0)
Any serious AE, n (%)	1 (0.8)	0 (0.0)	0 (0.0)	0 (0.0)
Any severe AE, n (%)	6 (5.1)	6 (5.0)	11 (9.2)	16 (13.6)
Any AE leading to study discontinuation^a^, n (%)	4 (3.4)	5 (4.2)	9 (7.5)	17 (14.4)
Any AE with probably or possible relationship to study drug, n (%)	5 (4.2)	9 (7.6)	10 (8.3)	12 (10.2)
Any AE leading to death, n (%)	0 (0.0)	0 (0.0)	0 (0.0)	0 (0.0)
**Treatment-emergent AEs reported for >2% of patients in any treatment group, n (%)**				
Infections and infestations	20 (16.9)	27 (22.7)	14 (11.7)	15 (12.7)
Upper respiratory tract infection	7 (5.9)	6 (5.0)	3 (2.5)	4 (3.4)
Nasopharyngitis	3 (2.5)	9 (7.6)	3 (2.5)	3 (2.5)
Urinary tract infection	3 (2.5)	3 (2.5)	1 (0.8)	1 (0.8)
Respiratory, thoracic and mediastinal disorders	8 (6.8)	8 (6.7)	14 (11.7)	17 (14.4)
Asthma^b^	3 (2.5)	4 (3.4)	9 (7.5)	14 (11.9)
Cough	4 (3.4)	2 (1.7)	1 (0.8)	1 (0.8)
Nervous system disorders	5 (4.2)	10 (8.4)	6 (5.0)	11 (9.3)
Headache	2 (1.7)	6 (5.0)	3 (2.5)	9 (7.6)
Gastrointestinal disorders	3 (2.5)	5 (4.2)	3 (2.5)	5 (4.2)
Diarrhoea	2 (1.7)	0 (0.0)	1 (0.8)	3 (2.5)

AEs = adverse events; N = total number of patients; n = number of patients in specified category; b.i.d. = twice daily.

Adverse events were coded using MedDRA™ version 9.0. At each level of summation (preferred term, system organ class and overall), each patient was counted only once. Percentages were based on the number of patients in the population for each treatment group.

a. Six of the 35 patients (1 fluticasone/formoterol, 1 fluticasone, 1 formoterol, and 3 placebo) had treatment-emergent adverse events reported as the primary reason for early discontinuation from the study. The remaining 29 patients (3 fluticasone/formoterol, 4 fluticasone, 8 formoterol, and 14 placebo) had lack of efficacy reported as the primary reason for early discontinuation from the study. A serious adverse event was one which resulted in death, was life threatening, resulted in persistent or significant disability/incapacity, inpatient hospitalisation or prolongation of existing hospitalisation, congenital anomaly or birth defect, or an important medical event.

b. An exacerbation of asthma was considered as an adverse event if it did not resolve with the study drug, including rescue albuterol/salbutamol, and additional medication was required (e.g., systemic glucocorticosteroids).

The most common adverse event leading to premature discontinuation of treatment in any group was asthma (fluticasone/formoterol combination therapy group, 2.5%; fluticasone group, 3.4%; formoterol group, 6.7%, placebo group, 11.9%). The most frequently reported adverse events occurring in more than 2% of patients in any treatment group are summarised in Table 5. There were no incidences of oropharyngeal candidiasis or dysphonia in any of the treatment groups. In addition, there were no clinically relevant changes or group differences for laboratory values (including glucose and potassium), vital signs, or ECG parameters.

## Discussion

The study presented here evaluated the efficacy and safety of fluticasone/formoterol 100/10 μg b.i.d. combination therapy compared to the individual components administered separately and placebo over a 12-week treatment period. The patients who took part in the study were adolescents and adults with mild-to-moderate asthma who were either already on ICS medication (either with or without a LABA) or who were ICS-free prior to screening.

The three co-primary endpoints all demonstrated that the fluticasone/formoterol combination product was superior in efficacy compared to each of the comparators. The first two co-primary efficacy endpoints evaluated lung function, based on FEV_1_ measurements, and compared the combination product with fluticasone alone and with formoterol alone, respectively. The improvements in FEV_1_ from pre-dose at baseline to pre-dose and 2 hours post-dose at week 12 were clinically relevant for patients receiving fluticasone/formoterol. Moreover, improvements seen in the combination therapy group were numerically and statistically significantly greater for the combination product compared with the monotherapies administered alone. Tachyphylaxis has been reported with formoterol monotherapy [37] and should be considered when interpreting the results. Nonetheless, fluticasone/formoterol combination therapy provided improvements from baseline in lung function over 12 weeks of treatment in this study. This improvement was seen throughout the study period, as shown by pulmonary function tests at weeks 2, 4, 8, and 12, and were supported by the secondary efficacy endpoints evaluating lung function, for example the morning and evening PEFR measurements.

The third co-primary endpoint, evaluated for the fluticasone/formoterol versus placebo treatment arms, showed that the combination product was statistically significantly superior to placebo with respect to the time to discontinuation due to lack of efficacy. Fewer patients in the combination therapy group prematurely left the study because of lack of treatment efficacy compared to those in any of the other three treatment groups.

Probably the most important and clinically relevant endpoint for patients is disease control. This was evaluated in this study by analysing the number of asthma control days, asthma exacerbations, rescue medication-free days, symptom-free days, and awakening-free nights. Although not a validated endpoint, the definition of asthma control used in this study was robust and highly relevant (recorded as days with no asthma symptoms, no sleep disturbance due to asthma, and no use of rescue medication). The use of the gatekeeping approach meant that these secondary endpoints could not be subjected to confirmatory statistical testing. However, the greatest improvements in these symptomatic endpoints were seen for patients administered the combination product compared to those receiving either of the individual treatments or placebo. Patients treated with the combination product also reported fewer asthma exacerbations throughout the study compared to each of the other treatment arms.

A potential criticism of this study could be the recruitment of patients who were not on ICS monotherapy at baseline, perhaps suggesting the potential for over-treatment of patients with milder asthma. This would not be consistent with GINA guidelines, which suggest a stepwise treatment approach whereby patients with persistent asthma should be initiated on a low to medium dose of inhaled corticosteroids prior to treatment escalation in the event of a suboptimal response. Nonetheless, the median FEV_1_ as a percentage of predicted value at baseline was relatively low (72–75% across the treatment groups), suggesting that a notable proportion of patients had moderately severe asthma and significant pulmonary impairment.

Furthermore, whilst the authors would not advocate a change to established treatment paradigm, two observations are nonetheless evident from this study. Firstly, pre-specified subgroup analysis of the populations of patients with no history of ICS use at baseline and those who were on an ICS at baseline demonstrate that both subgroups showed improvements for the two co-primary lung function endpoints over 12 weeks of treatment. Secondly, the treatment benefits for ICS-requiring patients were similar to or better than those for ICS-naïve patients on all three co-primary endpoints (versus the relevant comparator). These data may reflect the clinical benefit that patients could receive when stepping-up treatment to combination therapy.

Fluticasone/formoterol combination therapy demonstrated a good safety profile and was well-tolerated during the 12-week treatment period. There were no deaths or asthma exacerbations requiring hospitalisation in this study, and the adverse event profile of the combination therapy was similar to that of its individual components administered separately and placebo.

This study provides strong evidence of the benefits of a new combination of fluticasone/formoterol, administered via a single aerosol inhaler, in adolescent and adult patients with mild-to-moderate asthma.

## Conclusions

The data presented here are consistent with those observed in previous studies exploring the benefits of this ICS/LABA combination therapy [27,28]. The co-administration of formoterol and fluticasone shows superiority to the individual components administered separately and placebo for all three co-primary endpoints and confers significant benefits in terms of lung function, disease control, and asthma symptoms. This study therefore demonstrates that fluticasone/formoterol combination therapy is an efficacious and well tolerated treatment for asthma.

## Competing interests

RAN has received grant/research support from Abbott, Alcon, Amgen, AstraZeneca, Berringer-Ingleheim, Ception, Dey, Dyax, Genentech, GlaxoSmithKline, MAP, MedImmune, Novartis, Sanofi-Aventis, Sepracor, Shire, and TEVA; has been a consultant/scientific advisor for Genentech, GlaxoSmithKline, Merck, Novartis, ADVISOR and TEVA, and participated in a speaker’s bureau for AstraZeneca, Genentech, GlaxoSmithKline, MERCK, Novartis, Sanofi-Aventis, and UCB. VB had no proprietary interest in the tested product which was subject of the study and did not receive any payment from the sponsor (excluding the costs of conducting the study). ADU has received research, consulting and lecturing fees from GlaxoSmithkline, Sepracor, Schering Plough, Altana, Methapharma, Roche, AstraZeneca, Nycomed, ONO pharma, Novartis, and KOS Pharmaceuticals. KK was an employee of SkyePharma, which sponsored this study.

## Authors' contributions

RAN was the Principal Investigator for the study and participated in study design and conduct, ADU and VB were Investigators contributing to the conduct of the study, and KK participated in the study design and coordination for the Sponsor. All of the authors revised the manuscript critically for important intellectual content and read and approved the final manuscript.

## Pre-publication history

The pre-publication history for this paper can be accessed here:

http://www.biomedcentral.com/1471-2466/12/67/prepub
